# Neoadjuvant chemotherapy followed by hyperthermic intraperitoneal chemotherapy for patients with colorectal peritoneal metastasis: a retrospective study of its safety and efficacy

**DOI:** 10.1186/s12957-021-02255-w

**Published:** 2021-05-17

**Authors:** Sicheng Zhou, Yujuan Jiang, Jianwei Liang, Wei Pei, Zhixiang Zhou

**Affiliations:** Department of Colorectal Surgery, National Cancer Center/National Clinical Research Center for Cancer/Cancer Hospital, Chinese Academy of Medical Sciences and Peking Union Medical College, No. 17 Panjiayuan Nanli, Chaoyang District, Beijing, 100021 China

**Keywords:** Cytoreductive surgery, Hyperthermic intraperitoneal chemotherapy, Colorectal cancer, Peritoneal metastases, Neoadjuvant chemotherapy

## Abstract

**Background:**

Cytoreductive surgery (CRS) and hyperthermic intraperitoneal chemotherapy (HIPEC) are effective routine treatments for colorectal peritoneal metastasis (PM). However, the safety and efficacy of neoadjuvant chemotherapy (NAC) before CRS+HIPEC are poorly understood. Therefore, this study aimed to assess the perioperative safety and long-term efficacy of NAC prior to CRS+HIPEC for patients with synchronous colorectal PM.

**Methods:**

Patients with synchronous colorectal PM who received NAC prior to CRS+HIPEC were systematically reviewed at the China National Cancer Center and Huanxing Cancer Hospital from June 2017 to June 2019. The clinicopathologic characteristics, perioperative parameters, and survival rates of patients who underwent CRS+HIPEC with NAC (NAC group) and patients who underwent CRS+HIPEC without NAC (non-NAC group) were compared.

**Results:**

The study enrolled 52 patients, with 20 patients in the NAC group and 32 in the non-NAC group. In the NAC group, the proportion of patients with a peritoneal carcinomatosis index (PCI) score < 12 was significantly higher than that in the non-NAC group (80.0% vs 50.0%, *P* = 0.031), and more patients achieved complete cytoreduction (80.0% vs 46.9%, *P* = 0.018). The two groups had comparable grade III/IV complications and similar reoperation and mortality rates (*P* > 0.05). However, patients who received NAC had lower platelet counts (151.9 vs 197.7 × 10^9^/L, *P* = 0.036) and neutrophil counts (4.7 vs 7.2 × 10^9^/L, *P* = 0.030) on postoperative day 1. More patients survived for 2 years in the NAC group than in the non-NAC group (67.4% vs 32.2%, respectively, *P* = 0.044). However, the completeness of cytoreduction score (HR, 2.99; 95% CI, 1.14–7.84; *P* = 0.026), rather than NAC, was independently associated with overall survival (OS) in the multivariate analysis after controlling for confounding factors.

**Conclusion:**

NAC administration before CRS+HIPEC can be regarded as safe and feasible for patients with colorectal PM with comparably low mortality rates and acceptable morbidity rates. Nevertheless, large-sample randomized controlled studies are needed to confirm whether the administration of NAC before CRS+HIPEC confers a survival benefit to patients.

## Introduction

The peritoneum is the second most common site of colorectal cancer (CRC) metastasis after the liver [1], and 5–15% of CRC patients exhibit evidence of synchronous peritoneal metastasis (PM) [2, 3]. PM is well known to be an indicator of poor prognosis, and the median survival time of patients with PM is only 5–7 months [4]. Cytoreductive surgery (CRS) combined with hyperthermic intraperitoneal chemotherapy (HIPEC) has been carried out at experienced centres for select patients after extensive exploration and has become the cornerstone therapeutic strategy aiming to cure PM of CRC origin [5–7]. For selected patients, the median survival can reach approximately 40 months after CRS and HIPEC treatment for PM arising from CRC [8].

CRS+HIPEC is a complicated and potentially life-threatening procedure, with postoperative complication rates as high as 37.9–60.5% [9–14], which can worsen general conditions and hamper subsequent systemic adjuvant chemotherapy. Therefore, systemic adjuvant chemotherapy should be arranged before surgery whenever possible. Nevertheless, neoadjuvant chemotherapy (NAC) has been shown to benefit the survival of patients with stage IV CRC [15]. Relevant studies have also demonstrated that NAC before CRS+HIPEC is safe for CRC patients with PM and can provide certain survival benefits [16, 17]. On this basis, we performed a single-centre retrospective study to investigate the survival benefits of NAC prior to CRS+HIPEC as well as its perioperative safety for CRC patients with PM.

## Patients and methods

The present study included 52 eligible patients with synchronous PM arising from CRC who underwent CRS+HIPEC at the National Cancer Center and Huanxing Cancer Hospital between June 2017 and June 2019. The inclusion criteria included the following: (1) pathologically confirmed CRC, (2) aged between 18 and 75 years, and (3) Eastern Cooperative Group (ECOG) score ≤ 1. The exclusion criteria were as follows: (1) the presence of other malignant tumours, (2) palliative surgery such as bypass surgery or simple ostomy, (3) emergency operation, and (4) NAC administration for fewer than 3 cycles. The study protocol was approved by the Ethics Committee of the Cancer Hospital at the Chinese Academy of Medical Sciences (NCC 2017-YZ-026, Oct 17, 2017).

Patients were divided into two groups: those who received NAC followed by CRS+HIPEC (NAC group) and those who underwent CRS+HIPEC first without NAC (non-NAC group). Preoperative demographic and clinical information from the two groups was prospectively collected into an institutional database and retrospectively analysed. The treatment strategies for each patient were determined based on their wishes by multidisciplinary team meetings that incorporated radiologists and medical and surgical oncologists. In principle, CRS+HIPEC is typically used first for patients with resectable PM according to National Comprehensive Cancer Network (NCCN) guidelines. Conversely, systemic chemotherapy is often used for patients with extensive systemic metastases not subjectable to CC0-1 resection. The peritoneal carcinomatosis index (PCI) was used to assess the degree of PM, which was scored from 0 to 3 for each of the 13 defined areas of the abdominal cavity [18]. Intraoperative laparoscopic exploration was performed to calculate the PCI scores after NAC. The completeness of cytoreduction (CC) score was recorded as follows: CC 0/1, complete cytoreduction (CC 0 indicates no visible disease and CC 1 indicates nodules smaller than 0.25 cm); and CC 2/3, incomplete cytoreduction (CC 2 indicates a nodule size between 0.25 and 2.5 cm and CC 3 indicates nodules larger than 2.5 cm) [19]. Toxicity indexes of chemotherapy (blood, liver, and kidney toxicity), including the neutrophil count, platelet count, ALT level, and creatinine level, were measured in the morning on postoperative days (PODs) 1, 3, and 5. Postoperative complications were recorded and staged according to the Common Terminology Criteria for Adverse Events (CTCAE) classification within 30 days [20].

## CRS+HIPEC procedure

The CRS+HIPEC procedure has been described previously [21]. All patients received HIPEC via a closed technique after cytoreduction and to fashioning of the intestinal anastomoses. Oxaliplatin (200 mg/m^2^) and raltitrexed (3 mg/m^2^) with or without lobaplatin (50 mg/m^2^) were used for intraperitoneal chemotherapy. All patients were treated with a mixed solution of chemotherapeutic agents and 3 L of saline solution in the abdominal and pelvic cavities for 60 min at 42–43 °C. Thereafter, four catheters remained in the original position, and two more HIPEC procedures with the same chemotherapeutic regimens as well as perfusion times were reperformed on the second and fourth days after surgery in the ward. CRS+HIPEC treatment was performed by two surgical specialists with more than 20 years of experience in gastrointestinal surgery at the two centres; the exact same HIPEC technique and postoperative treatment were performed at both centres.

## Follow-up

All patients were scheduled to receive follow-up through outpatient visits every 3 months for the first 3 years and then every 6–12 months for the 3 years thereafter. CT scans of the abdomen and pelvis and laboratory examinations, including those of tumour biomarkers (CEA and CA 19–9), were performed at every follow-up. The long-term endpoint of this study was the 3-year overall survival (OS) rate. OS was defined as the time from the date of surgery until death or the last follow-up (July 31, 2020).

## Statistical analysis

All variables were compared between the groups using IBM SPSS Statistics software version 24.0 (IBM Corp, Armonk, NY, USA). Continuous variables are presented as the mean ± SD and were analysed with Student’s *t* tests or Mann-Whitney *U* tests depending on the distribution. Differences between fractions were analysed by *χ*^2^ tests or Fisher’s exact tests as appropriate. Actuarial OS was estimated by the Kaplan-Meier method. Univariate analysis of variables potentially impacting OS was performed with the log-rank test, and significant univariate variables were applied in the multivariate Cox regression model. A *P* value lower than 0.05 was regarded as statistically significant.

## Results

In the present study, 52 patients with synchronous PM arising from CRC underwent CRS+HIPEC; 20 (38.5%) received NAC, and 32 (61.5%) underwent surgery without receiving NAC. Patients in the NAC group were treated with a chemotherapy regimen commonly used for CRC, and 6 patients received the antiangiogenic agent bevacizumab, as shown in Table 1. The patient demographics and clinical details are provided in Table 2. In the NAC group, 80.0% of the patients achieved complete cytoreduction (CC 0/1), versus 46.9% in the non-NAC group (*P* = 0.018). In addition, the proportion of patients with a PCI score < 12 in the NAC group was significantly higher than that in the non-NAC group (80.0% vs 50.0%, *P* = 0.031). There were no significant differences between the two groups in terms of age, sex, body mass index, preoperative CEA level, comorbidity, tumour location, histology, T stage, N stage, liver metastases, ascites, HIPEC regimen, or adjuvant chemotherapy.

**Table 1:** Neoadjuvant chemotherapy regimens for 20 patients

Preoperative chemotherapy regimens	*N* (%)
XELOX	5 (25.0)
XELOX+bevacizumab	2 (10.0)
FOLFOX	5 (25.0)
FOLFIRI	3 (15.0)
FOLFOX+bevacizumab	2 (10.0)
FOLFIRI+bevacizumab	2 (10.0)
5-FU+leucovorin	1 (5.0)

*XELOX* capecitabine+oxaliplatin, *FOLFOX* leucovorin calcium+5-fluorouracil+oxaliplatin, *FOLFIRI* leucovorin calcium+5-fluorouracil+irinotecan

**Table 2:** Clinical characteristics of the 52 enrolled patients

Variable	All patients (*n* = 52)	NAC (*n* = 20)	Non-NAC (*n* = 32)	*P*
Age (years)				0.350
< 65	35 (67.3)	15 (75.0)	20 (62.5)	
65	17 (22.7)	5 (25.0)	12 (37.5)	
Gender				0.508
Male	29 (55.8)	10 (50.0)	19 (59.4)	
Female	23 (44.2)	10 (50.0)	13 (40.6)	
Body mass index (kg/m^2^)	22.5 ± 3.5	22.8 ± 3.3	22.4 ± 3.6	0.542
Preoperative CEA level (ng/ml)				0.744
< 5	17 (32.7)	6 (30.0)	11 (34.4)	
≥ 5	35 (67.3)	14 (70.0)	21 (65.6)	
Comorbidity	14 (26.9)	4 (20.0)	10 (31.3)	0.374
Tumour location				0.636
Colon	42 (80.8)	15 (75.0)	27 (84.4)	
Rectum	10 (19.2)	5 (25.0)	5 (15.6)	
Histology				0.289
Adenocarcinoma	29 (55.8)	13 (65.0)	16 (50.0)	
Mucinous/signet-ring	23 (44.2)	7 (35.0)	16 (50.0)	
T stage				0.287
T3	6 (11.5)	4 (20.0)	2 (6.3)	
T4	46 (88.5)	16 (80.0)	30 (93.7)	
N stage				0.506
N0	8 (15.4)	3 (15.0)	5 (15.6)	
N1	16 (30.8)	8 (40.0)	8 (25.0)	
N2	28 (53.8)	9 (45.0)	19 (59.4)	
PCI score				0.031
< 12	32 (61.5)	16 (80.0)	16 (50.0)	
≥ 12	20 (38.5)	4 (20.0)	16 (50.0)	
PCI score	11.9 ± 5.6	9.8 ± 4.7	13.7 ± 6.4	0.006
Liver metastases	9 (17.3)	4 (20.0)	5 (15.6)	0.977
Ascites	22 (42.3)	8 (40.0)	14 (43.8)	0.790
HIPEC regimen				0.930
Lobaplatin+oxaliplatin+raltitrexed	23 (44.2)	9 (45.0)	14 (43.8)	
Oxaliplatin+faltitrexed	29 (55.8)	11 (55.0)	18 (56.2)	
CC score				0.018
0–1	31 (59.6)	16 (80.0)	15 (46.9)	
2–3	21 (40.4)	4 (20.0)	17 (53.1)	
Adjuvant chemotherapy				0.738
Yes	44 (84.6)	16 (80.0)	28 (87.5)	
No	8 (15.4)	4 (20.0)	4 (12.5)	

*PCI* peritoneal carcinomatosis index, *CC* cytoreduction score

Table 3 lists the surgical outcomes and postoperative course. The mean operative time in the NAC group was shorter than that in the non-NAC group, but the difference was not statistically significant (245.5 vs 289.4 min, *P* = 0.082). With regard to toxicity indexes after HIPEC, patients in the NAC group were more likely to experience thrombocytopenia after HIPEC than those in the non-NAC group (30.0% vs 6.3%, *P* = 0.043). There were no significant differences in the rates of grade III/IV complications (40.0% vs 31.3%, *P* = 0.519), hospital stay length (15.4 vs 13.9 days, *P* = 0.333), or 30-day reoperation rate (1.9% vs. 0%, *P* = 1.000) between patients in the NAC and non-NAC groups.

**Table 3:** Surgical outcomes and postoperative courses of the 52 enrolled patients

Variable	All patients (*n* = 52)	NAC (*n* = 20)	Non-NAC (*n* = 32)	*P*
Operative time (min, mean ± SD)	276.3 ± 60.1	245.5 ± 59.1	289.4 ± 63.4	0.082
Intraoperative blood loss (mL, mean ± SD)	104.3± 111.1	115.0 ± 111.3	100.8 ± 111.0	0.661
Postoperative complication (Grades 3–4)	18 (34.6)	8 (40.0)	10 (31.3)	0.519
Arrhythmia	1 (1.9)	1 (5.0)	0 (0)	
Pneumonia	3 (5.8)	2 (10.0)	1 (3.1)	
Anastomotic leakage	3 (5.8)	1 (5.0)	2 (6.3)	
Ileus	7 (13.5)	3 (15.0)	4 (12.5)	
Abdominal abscess	3 (5.8)	1 (5.0)	2 (6.3)	
Intra-abdominal haemorrhage	1 (1.9)	0 (0)	1 (3.1)	
Wound infection	5 (9.6)	2 (10.0)	3 (9.4)	
Toxicity indexes after HIPEC	22 (42.3)	10 (50.0)	12 (37.5)	0.375
Abnormal changes in neutrophils	6 (11.5)	3 (15.0)	3 (9.4)	0.864
Abnormal changes in platelets	8 (15.4)	6 (30.0)	2 (6.3)	0.043
Abnormal changes in ALT	8 (15.4)	2 (10.0)	6 (18.8)	0.463
Abnormal changes in creatinine	3 (5.8)	1 (5.0)	2 (6.3)	1.000
Time to first flatus (day, mean ± SD)	3.8 ± 1.8	4.1 ± 1.9	3.6 ± 1.8	0.811
Total hospital stay (day, mean ± SD)	14.4 ± 5.0	15.4 ± 6.0	13.9 ± 3.9	0.333
Re-operation	1 (1.9)	1 (5.0)	0 (0)	1.000
Mortality (%)	0 (0)	0 (0)	0 (0)	1.000

**Fig. 1:**
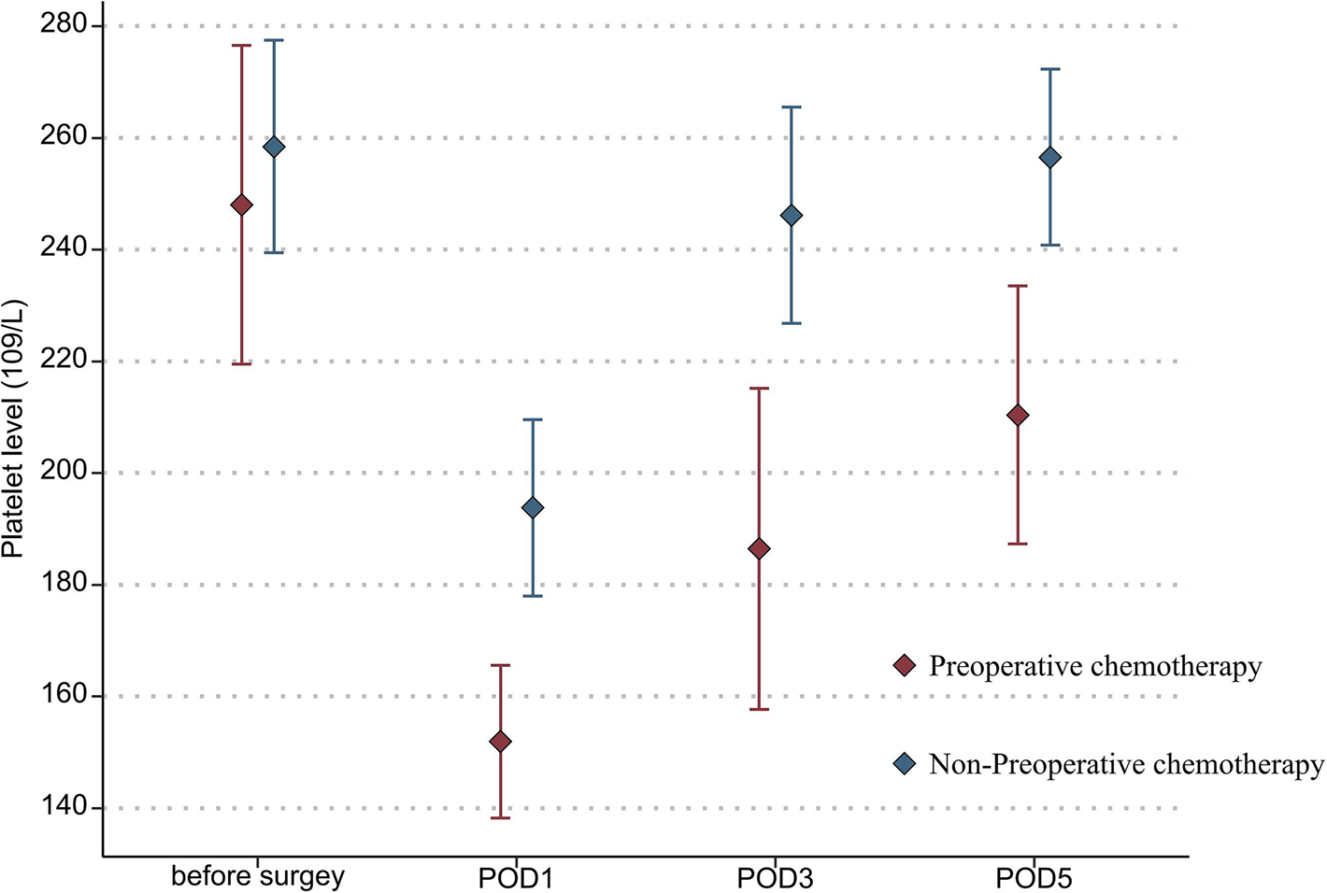
Mean platelet levels in the two groups of patients before surgery and from postoperative days 1 through 5 (statistical comparison made by Student’s *t* test)

**Fig. 2:**
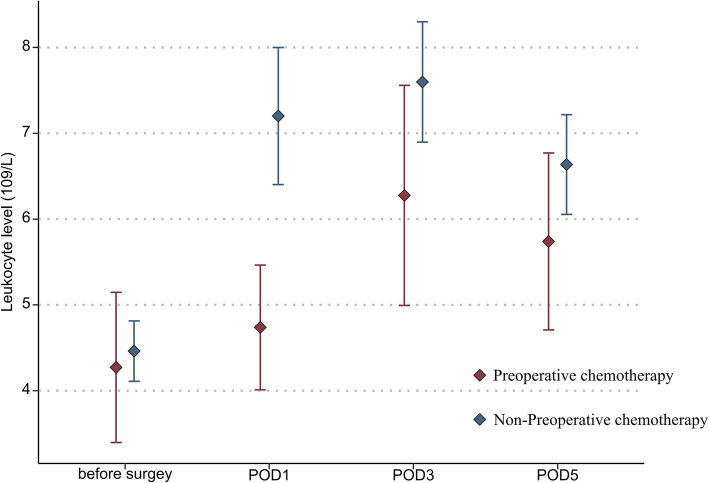
Mean leukocyte levels in the two groups of patients before surgery and from postoperative days 1 through 5 (statistical comparison made by Student’s *t* test)

**Fig. 3:**
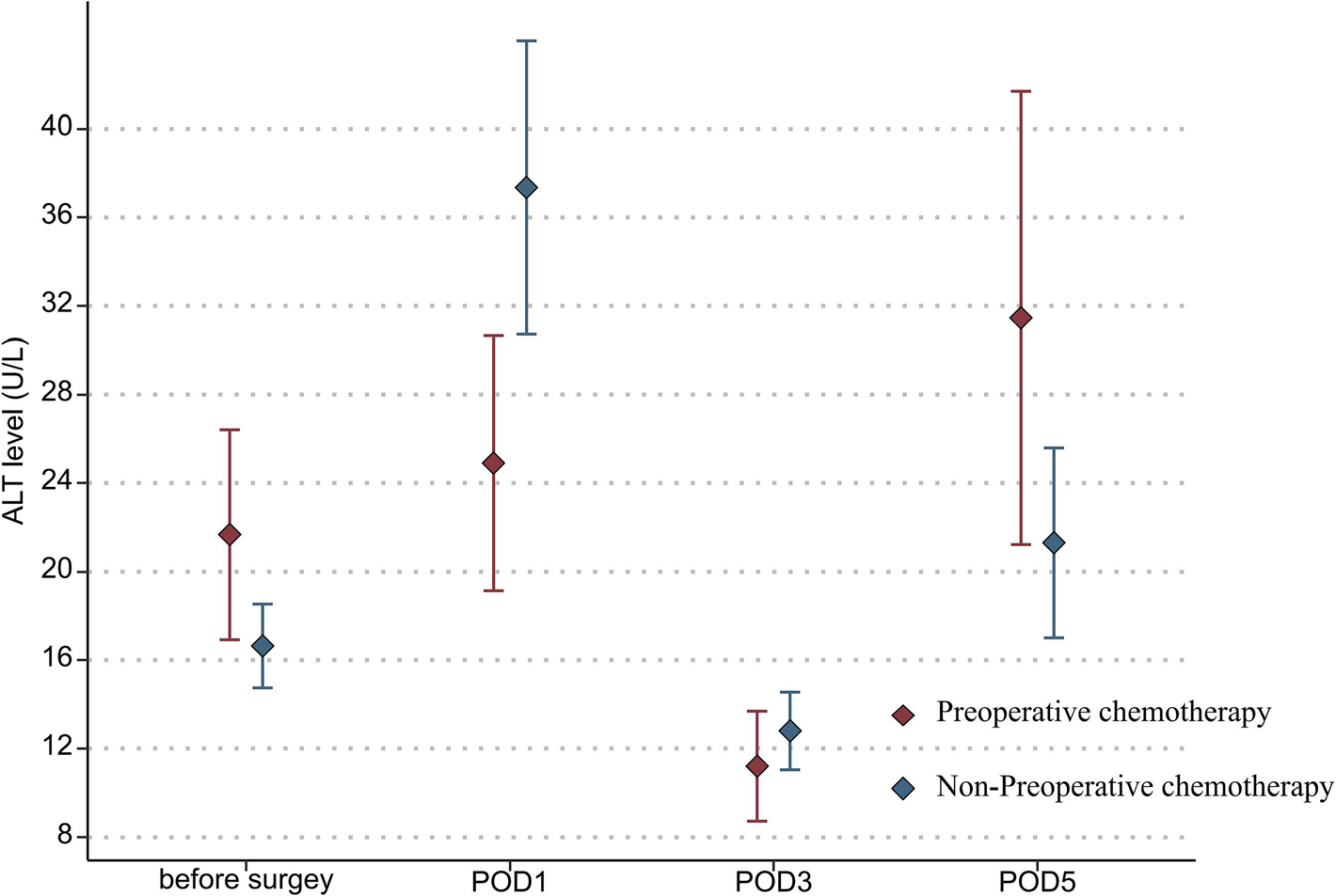
Mean ALT levels in the two groups of patients before surgery and from postoperative days 1 through 5 (statistical comparison made by Student’s *t* test)

**Fig. 4:**
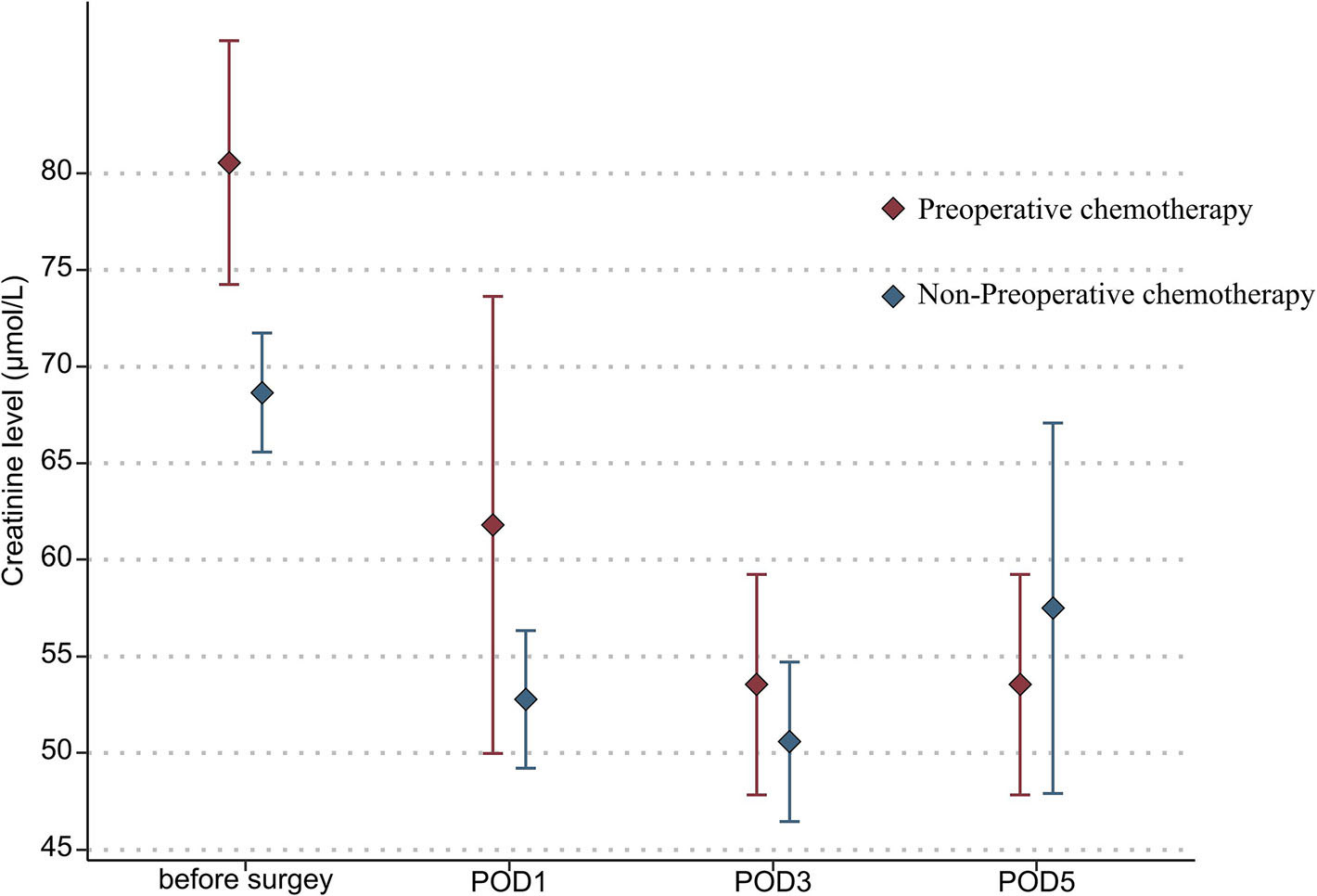
Mean creatinine levels in the two groups of patients before surgery and from postoperative days 1 through 5 (statistical comparison made by Student’s *t* test)

In addition, the mean platelet count (151.9 vs 197.7 × 10^9^/L, *P* = 0.036) and leukocyte count (4.7 vs 7.2 × 10^9^/L, *P* = 0.030) in the NAC group were also significantly lower than those in the non-NAC group on POD 1 (Fig. 1 and Fig. 2). The ALT and creatinine levels on PODs 1, 3, and 5 were not statistically different between the two groups (Fig. 3 and Fig. 4).

## Survival analysis

**Fig. 5:**
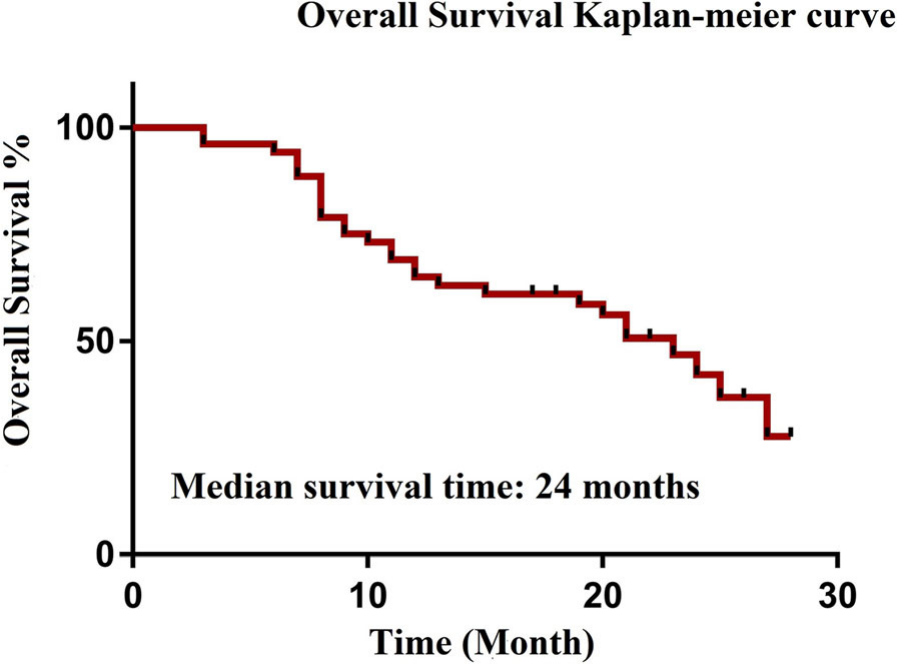
Overall survival rates of 52 patients who underwent CRS+HIPEC after NAC

**Fig. 6:**
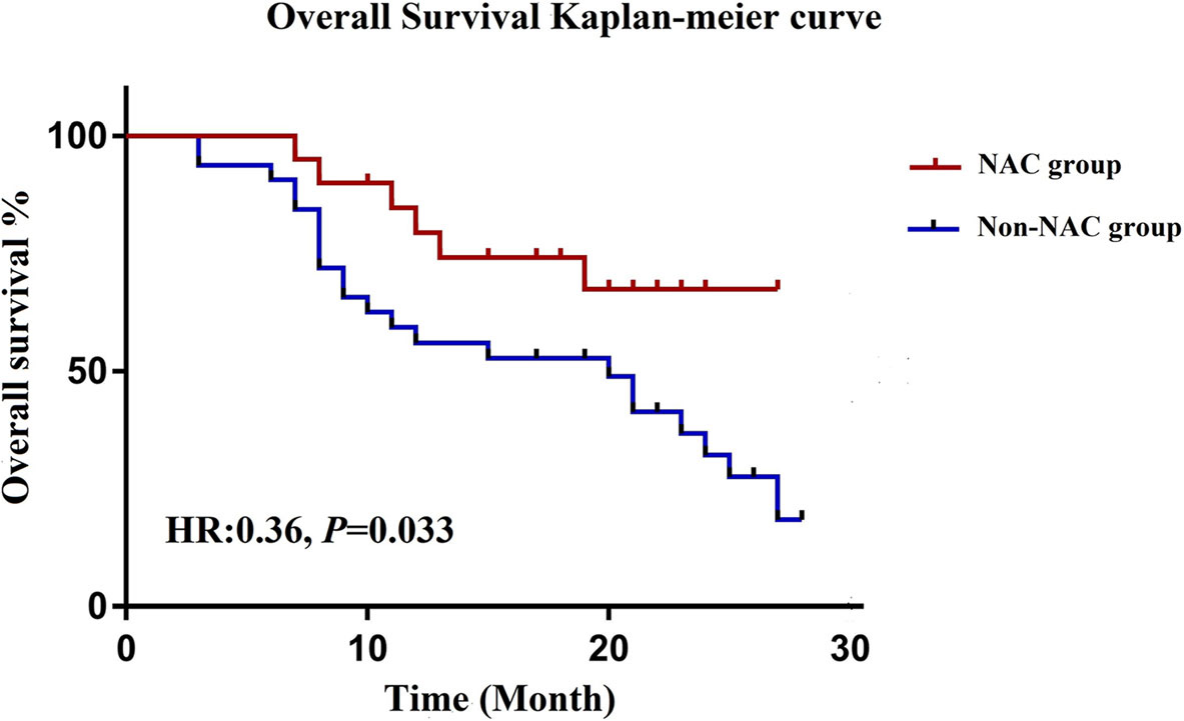
Overall survival curves for the NAC and non-NAC groups (statistical comparison made by the log-rank test)

The median follow-up time was 18.5 (range, 3–28) months, and 3 patients were lost to follow-up. During the follow-up period, 28 patients died of tumour recurrence and metastasis, including 6 in the NAC group and 22 in the non-NAC group. The median survival for all patients was 24 months, and the estimated 1- and 2-year OS rates for the entire cohort were 65.0% and 42.1%, respectively (Fig. 5). Patients who received NAC prior to CRS+HIPEC had a higher 2-year OS rate than those who underwent CRS+HIPEC without NAC (32.2% vs 67.4%, *P* = 0.044) (Fig. 6).

The results of exploratory univariate and multivariate Cox regression analyses of OS are detailed in Table 4. The univariate analysis identified the following prognostic indicators for OS: neoadjuvant chemotherapy (HR, 0.36; 95% CI, 0.13–0.92; *P* = 0.033), the PCI score (HR, 2.98; 95% CI, 1.41–6.32; *P* = 0.004), and the CC score (HR, 4.20; 95% CI, 1.89–9.36; *P* < 0.001). After correction for these variables in the multivariate analysis, OS was significantly associated with the CC score (HR, 2.99; 95% CI, 1.14–7.84; *P* = 0.026). Neoadjuvant chemotherapy (HR, 0.55; 95% CI, 0.22–1.39; *P* = 0.204) and the PCI score (HR, 1.49; 95% CI, 0.61–3.66; *P* = 0.381) were not independently associated with OS.

**Table 4:** Univariate and Multivariable Cox Regression Analysis for Overall Survival

Variable	Overall survival			
	Univariate analysis		Multivariate analysis	
	HR (95%CI)	*P*	HR (95%CI)	*P*
Gender: female/male	1.13 (0.53–2.43)	0.753		
Age at operation (≥ 65 years/< 65 years)	2.12 (0.98–4.60)	0.056		
Preoperative chemotherapy (yes/no)	0.36 (0.13–0.92)	0.033	0.55 (0.22–1.39)	0.204
T stage (T4/T3)	1.61 (0.73–3.54)	0.235		
N stage				
N0	Reference	Reference		
N1	1.29 (0.57–2.94)	0.545		
N2	2.26 (0.71–7.16)	0.167		
Site of original (rectum/colon)	1.48 (0.63–3.51)	0.373		
Histology (mucinous/adenocarcinoma)	2.05 (0.96–4.40)	0.065		
Preoperative CEA level (≥ 5 ng/ml/< 5 ng/ml)	1.68 (0.78–3.58)	0.183		
Liver metastases (yes/no)	1.24 (0.50–3.08)	0.648		
HIPEC regimen (lobaplatin/non-lobaplatin)	1.38 (0.62–3.05)	0.427		
Presence of ascites (yes/no)	1.20 (0.55–2.60)	0.650		
PCI score (≥ 12/< 12)	2.98 (1.41–6.32)	0.004	1.49 (0.61–3.66)	0.381
CC score (2–3/0–1)	4.20 (1.89–9.36)	< 0.001	2.99 (1.14–7.84)	0.026
Grade 3–4 postoperative complication (yes/no)	1.62 (0.74–3.57)	0.229		

## Discussion

In contrast to mucinous appendiceal neoplasms, PM from CRC is usually a manifestation of extensive tumour progression and poor prognosis. However, CRS+HIPEC can improve the long-term survival rates of well-selected patients with PM [5–8]. In addition to CRS+HIPEC treatment, perioperative systemic chemotherapy has been shown to prolong the survival of patients with PM [15, 16, 22]. However, the optimal timing of chemotherapy, especially the safety and long-term efficacy of preoperative chemotherapy, remains unknown. Therefore, this study was conducted to investigate the perioperative safety and survival benefits of performing NAC before the CRS+HIPEC procedure for CRC patients with synchronous PM. Our data revealed that patients who underwent CRS+HIPEC following NAC had a comparably low mortality rate and an acceptable morbidity rate. Although NAC has a certain effect on platelets and neutrophils, these responses did not appear to translate to postoperative complications under close supervision and active surveillance. Nevertheless, while patients who underwent NAC experienced improvements in OS compared to those who underwent only surgery, NAC was not an independent prognostic factor for improving OS after controlling for confounding factors. However, the selection of chemotherapy drugs in this study lacked a unified standard, and targeted drugs such as bevacizumab were adopted in some patients, which potentially interfered with the safety and oncological treatment efficacy of NAC.

CRS+HIPEC is a complex and potentially life-threatening procedure that has a morbidity rate ranging from 12 to 52% and a mortality rate ranging from 0.9 to 5.8% at 10 specified international treatment centres [23]. Therefore, it is worth exploring whether the side effects of NAC, such as myelosuppression, neurotoxicity, and gastrointestinal reactions, further increase morbidity and mortality rates after CRS+HIPEC. The retrospective study of Devilee et al. assessed the safety and efficacy of NAC for 91 patients undergoing CRS and HIPEC, and the results showed that NAC did not significantly increase the incidence of severe complications (24% vs 17%, *P* = 0.55) or mortality (0 vs 1.5%, *P* = 1.000) [24]. Similarly, Leimkühler et al. observed that the severity and timing of complications were comparable between patients who received NAC and those who did not [17]. Our study also found that patients who underwent NAC were more likely to experience thrombocytopenia after surgery (30.0% vs 6.3%, *P* = 0.043). Moreover, the mean platelet count (151.9 vs 197.7 × 10^9^/L, *P* = 0.036) and neutrophil count (4.7 vs 7.2 × 10^9^/L, *P* = 0.030) in the NAC group were significantly lower than those in the non-NAC group on POD 1. However, we found that these adverse effects on platelets and neutrophils did not appear to translate into severe postoperative complications (40.0% vs 31.3%, *P* = 0.519).

In theory, NAC can effectively downgrade the primary tumour and peritoneal tumour burden and thus improve long-term survival. In the present study, patients who underwent NAC had significantly lower PCI scores at the time of CRS+HIPEC than those who did not, and a higher proportion of these patients achieved complete cytoreduction (CC 0/1). Moreover, although the CC score, rather than NAC, was independently associated with OS in the multivariate analysis, more patients who received NAC survived for 2 years than those who did not (67.4% vs 32.2%, respectively, *P* = 0.044). Large-scale multi-institutional registry studies showed that both the PCI score and CC score were associated with long-term survival after CRS+HIPEC [25, 26], and the use of NAC to reduce the tumour burden and increase the chances of CC is an effective and valuable strategy. Consistent with these findings, a retrospective analysis of 298 patients conducted by Beal et al. demonstrated that NAC benefited the survival of patients undergoing CRS+HIPEC, although NAC was not identified as an independent factor for OS after controlling for confounding factors [13]. Similarly, Devilee et al. reported that treatment with NAC was associated with improved OS before CRS+HIPEC [24].

Several limitations of the current study should be taken into account. The most significant limitation was the small size, especially in the NAC group, which included only 20 patients; this might have been responsible for some of the observed differences between the two groups. Second, this study was retrospective and had inherent selection bias. Third, vital information, such as KRAS and BRAF mutations, was lacking in some patients, making it difficult to evaluate their prognostic values. Furthermore, the chemotherapy regimens of 20 patients who underwent NAC were not uniform, and some patients were administered targeted drugs such as bevacizumab, which interfered with the prognostic analysis. Finally, the follow-up time of this study was short (median of only 18.5 months), which artificially improved the prognosis. Multicentre, large-sample prospective randomized controlled studies are needed to further confirm our results.

In conclusion, the results of our study demonstrate that NAC prior to CRS+HIPEC had a comparably low mortality rate and an acceptable morbidity rate. Although NAC exerts certain effects on platelets and neutrophils, these effects do not appear to translate to severe postoperative complications. Nevertheless, large-sample randomized controlled studies are needed to confirm whether the administration of NAC before CRS+HIPEC confers a survival benefit to patients.

## Data Availability

While the patient data are confidential, they are available.

## Abbreviations

CRC: Colorectal cancer
PM: Peritoneal metastasis
CRS: Cytoreductive surgery
HIPEC: Hyperthermic intraperitoneal chemotherapy
NAC: Neoadjuvant chemotherapy
ECOG: Eastern Cooperative Group
PCI: Peritoneal carcinomatosis index
CC score: Completeness of cytoreduction score
POD: Postoperative day
CTCAE: Common Terminology Criteria for Adverse Events
OS: Overall survival
